# Microbiological and Clinical Predictors of Surgical Intervention in Odontogenic Sinusitis

**DOI:** 10.3390/jcm14207342

**Published:** 2025-10-17

**Authors:** So Jeong Kim, Jung Ho Bae

**Affiliations:** 1 Department of Otorhinolaryngology-Head and Neck Surgery, School of Medicine, Ewha Womans University Mokdong Hospital, Seoul 07985, Republic of Korea; entsj@ewha.ac.kr; 2 Department of Otorhinolaryngology-Head and Neck Surgery, School of Medicine, Ewha Womans University Seoul Hospital, Seoul 07804, Republic of Korea

**Keywords:** odontogenic sinusitis, oral and maxillofacial surgery, antibiotic resistance, microbiological profile, risk factors

## Abstract

**Background:** Odontogenic sinusitis (ODS) is a frequent but under-recognized cause of chronic maxillary sinusitis, often requiring multidisciplinary management. Understanding clinical and microbiological predictors for oral and maxillofacial surgery (OMS) treatment may aid in early risk stratification. **Methods:** We retrospectively reviewed 47 patients diagnosed with ODS at a tertiary referral center between January 2023 and April 2025. Clinical characteristics, dental pathologies, and microbiological findings were analyzed. Univariate and multivariate logistic regression analyses were performed to identify independent predictors of OMS intervention. **Results:** The cohort included 20 males and 27 females, with a mean age of 59.5 years. OMS intervention was performed for 21 patients (44.7%). These patients were younger, and more frequently presented with gingival pain or swelling, oroantral fistula, antibiotic-resistant organisms, and polymicrobial infections compared with the non-OMS group. *Staphylococcus epidermidis* was the most common isolate, followed by *Streptococcus constellatus*, *Klebsiella pneumoniae*, and *Pseudomonas aeruginosa*. Antibiotic resistance was observed in 29.8% of patients and was significantly more prevalent in the OMS group (52.4% vs. 7.7%, *p* < 0.001). Multivariate analysis identified dental diagnosis and antibiotic resistance as independent predictors for OMS intervention. All patients achieved full recovery following surgery, with no treatment failures. **Conclusions:** ODS demonstrates distinct clinical and microbiological characteristics, with antibiotic resistance and specific dental pathologies serving as independent risk factors for OMS intervention. Recognition of these features may guide early multidisciplinary planning and improve clinical outcomes.

## 1. Introduction

Chronic rhinosinusitis (CRS) is defined as inflammation of the nose and paranasal sinuses lasting for more than 12 weeks, characterized by two or more symptoms, one of which should be either nasal blockage/congestion or nasal discharge, with or without facial pain/pressure and/or reduction in smell, and either endoscopic signs or computed tomography (CT) findings of disease [1]. The prevalence of CRS in Europe is estimated to be 10.9% of the general population aged 15–75 years, representing a considerable healthcare burden [2]. In this manuscript, the term ‘oral and maxillofacial surgery (OMS)’ refers specifically to the combined surgical discipline involved in the multidisciplinary management of odontogenic sinusitis (ODS). ODS, a subtype of CRS, arises from infectious dental pathology or iatrogenic dental procedures [3]. It accounts for approximately 25–40% of chronic maxillary sinusitis and 45–75% of unilateral maxillary sinusitis on computed tomography, according to radiologic series from tertiary and community-based populations [3,4,5,6]. The condition typically results from disruption of the Schneiderian membrane by ascending dental infection, odontogenic cysts, or iatrogenic causes such as implant displacement, sinus lift complications, and other dental procedures [3]. The molars are most commonly implicated, with tooth extraction reported as a leading etiologic factor [7]. ODS is frequently under-recognized due to its non-specific presentation; however, compared to rhinogenic CRS, foul smell and purulent rhinorrhea are more common and more specific clinical features [6,8,9]. If inadequately treated, ODS may progress to serious complications involving the orbital or cranial cavity [3]. Microbiologically, ODS is generally a polymicrobial infection dominated by anaerobes, including *Peptostreptococcus*, *Prevotella*, *Fusobacterium*, and *viridans streptococci* [10,11,12]. In contrast, rhinogenic CRS more commonly involves *Streptococcus pneumoniae*, *Haemophilus influenzae*, and *Moraxella catarrhalis* [13]. This distinct microbiology highlights the importance of accurate diagnosis and appropriate treatment of the underlying dental pathology [14]. Because ODS originates from a dental source, definitive management should begin with elimination of the odontogenic focus through dental intervention—most commonly endodontic (root canal) treatment or extraction of a non-restorable tooth [1,15]. Antibiotics and nasal irrigation may provide temporary symptom relief but are considered adjunctive rather than curative measures, as resolution occurs in only 15–20% of cases when the dental source is untreated. Therefore, odontogenic sinusitis cannot be cured with antibiotics alone; complete resolution requires targeted management of the underlying dental pathology, often necessitating multidisciplinary collaboration between otolaryngologists and oral and maxillofacial surgeons. When dental or medical therapy is insufficient to control infection or restore sinus drainage, surgical intervention is warranted [16]. Optimal outcomes often require a multidisciplinary approach involving both otolaryngologists and oral and maxillofacial surgeons [17]. Given that odontogenic sinusitis frequently presents with diverse microbiological spectra and variable antibiotic resistance profiles, the timely recognition of high-risk patients remains challenging. The present study therefore aimed to investigate bacterial isolates, antibiotic resistance, and their associations with specific dental pathologies, and to determine independent predictors for OMS intervention. Our goal was to provide clinicians with evidence-based guidance to support early risk stratification and multidisciplinary decision-making in the management of odontogenic sinusitis.

## 2. Materials and Methods

### 2.1. Study Population

This retrospective study included patients diagnosed with odontogenic sinusitis (ODS) at a tertiary referral medical center between January 2023 and April 2025. A total of 79 patients were initially evaluated by chart review, and 47 patients met the eligibility criteria and were included in the final analysis. The diagnosis of ODS was established based on clinical symptoms, nasal endoscopy, paranasal sinus computed tomography (CT), and dental evaluation. Gingival pain and swelling were assessed jointly by the otolaryngology and dental teams during preoperative evaluation. Clinical findings were confirmed through intraoral examination by the dental specialist, supplemented by patient-reported symptoms and documentation in the shared medical–dental records.

Inclusion criteria were as follows: (1) age > 18 years, (2) radiologic evidence of chronic maxillary sinusitis confirmed on paranasal sinus CT, and (3) odontogenic origin, such as dental caries, periapical abscess, periodontal disease (only when accompanied by apical bone loss or sinus-floor disruption on CT), or implant-related pathology. Cases of isolated periodontal disease without radiologic evidence of bone involvement were excluded. All included patients had failed prior conservative treatment, including antibiotics, nasal saline irrigation, and dental interventions such as root canal treatment, periodontal debridement, or implant revision. Patients with ODS secondary to head and neck radiation therapy, maxillofacial trauma, sinonasal malignancy, current chemotherapy, pregnancy, alcohol or drug misuse, or prior sinus surgery were excluded.

### 2.2. Surgical Procedure

All surgeries were performed under general anesthesia by a single rhinologist. Preoperatively, each case was jointly reviewed with the OMS team, and OMS participation was planned when concurrent dental procedures (tooth extraction, implant/prosthesis removal, or oroantral fistula [OAF] repair) were required. In this study, OMS intervention was defined as surgical management of odontogenic pathology performed by oral and maxillofacial surgeons during or in conjunction with endoscopic sinus surgery. Standard endoscopic sinus surgery (ESS) was performed, including uncinectomy and posteroinferior enlargement of the natural maxillary ostium using a microdebrider. Diseased mucosa, polyps, or foreign materials (such as displaced implants or grafts) were removed while preserving healthy mucosa whenever possible.

When an OAF was present, closure was achieved using conventional methods such as buccal fat pad graft, mucosal advancement flap, or palatal flap. Additional procedures such as implant removal or sequestrectomy were performed when necessary.

### 2.3. Sample Collection

Samples were collected from all patients at the onset of surgery. Bacterial specimens were obtained through the surgically enlarged natural ostium of the maxillary sinus using a sterile microtip swab under endoscopic guidance. The specimens were immediately placed in Stuart’s transport medium and transported to the microbiology laboratory within 2 h. All samples were inoculated onto 5% sheep blood agar, chocolate agar, Columbia anaerobic blood agar, and thioglycolate broth, and incubated under aerobic, anaerobic, and microaerophilic conditions. Cultures were processed using standard microbiological techniques. Anaerobic cultures were incubated in an anaerobic chamber with 5% CO_2_, 10% H_2_, and 85% N_2_ for up to 7 days, as recommended by the ASM Manual of Clinical Microbiology and the Clinical and Laboratory Standards Institute (CLSI) guidelines. Antimicrobial susceptibility testing of the identified bacterial isolates was performed using the agar disk diffusion method according to CLSI standards, and resistance patterns were analyzed to characterize the prevalence of antibiotic-resistant organisms.

### 2.4. Treatment Outcome

All patients were followed postoperatively at 4 weeks and 12 weeks, and subsequently at 12-week intervals. The minimum follow-up duration was 6 months, with the longest observation period extending to 18 months. Cure was defined as a resolution of sinonasal and dental symptoms, absence of purulent rhinorrhea or postnasal drip on nasal endoscopy, and absence of abnormal findings on follow-up CT. Patients were evaluated at 12-week intervals during the outpatient follow-up.

### 2.5. Statistical Analysis

A post hoc power analysis was performed to assess whether the sample size was sufficient to detect clinically meaningful differences between groups. With an alpha level of 0.05, an assumed effect size (odds ratio) of 3.0—based on previously reported effect sizes (OR ≈ 2.5–3.5) for significant clinical predictors in odontogenic and sinonasal infection studies—and the observed group distribution (26 vs. 21 patients), the calculated power was 0.82, exceeding the conventional threshold of 0.80. This suggests that the sample size was adequate to identify significant predictors in the multivariate logistic regression model. Descriptive statistics summarized patient demographics, clinical characteristics, and microbiological findings. Continuous variables were analyzed using the independent *t*-test or the Mann–Whitney U test, depending on data distribution. Categorical variables were compared using Pearson’s chi-square test or Fisher’s exact test, as appropriate. Variables showing univariate *p* < 0.10 before false discovery rate (FDR) correction, together with clinically relevant factors, were entered into the multivariable logistic regression model. The FDR adjustment presented in Table 1 was applied only to exploratory descriptive comparisons, whereas the regression model was designed to identify potential independent associations. Odds ratios (ORs) with 95% confidence intervals (CIs) were reported. A two-tailed *p* < 0.05 was considered statistically significant. Model discrimination and calibration were assessed by the area under the receiver-operating characteristic curve (AUC) and the Hosmer–Lemeshow goodness-of-fit test (10 risk deciles). To address possible small-sample bias and quasi-separation (particularly for predictors such as implant protrusion), a ridge-penalized logistic regression was additionally performed as a sensitivity analysis. The penalized model demonstrated coefficient stability and consistent association directions, confirming the robustness of the main model. All analyses were performed using IBM SPSS Statistics, version 29.0 (IBM Corp., Armonk, NY, USA).

## 3. Results

A total of 47 patients diagnosed with ODS were included in this study, comprising 20 males (42.6%) and 27 females (57.4%). The mean age was 59.5 ± 15.1 years. According to OMS intervention status, 26 patients (55.3%) were classified into the non-OMS intervention group (Group A) and 21 patients (44.7%) into the OMS intervention group (Group B). Patients in Group A were significantly older than those in Group B (64.4 ± 14.8 vs. 53.5 ± 13.5 years, *p* = 0.012). Hypertension was more frequently observed in Group A compared to Group B (38.5% vs. 9.5%, *p* = 0.020). Gingival pain and swelling were reported more frequently in Group B (19.2% vs. 0.0%, *p* = 0.035). Other comorbidities, including diabetes mellitus, osteoporosis, cancer history, and hematologic disease, were rare and did not differ significantly between the groups. Regarding dental diagnoses, oroantral fistula was significantly more frequent in Group B compared with Group A (66.7% vs. 23.1%, *p =* 0.007). Implant protrusion into the sinus was significantly higher in Group A than in Group B (23.1% vs. 0.0%, *p =* 0.026). In contrast, periapical abscess, chronic periodontitis, and MRONJ showed no statistically significant differences between the two groups (Table 1). 

Bacterial pathogens were isolated from all patients. The most frequently identified species were *Staphylococcus epidermidis* (34.0%), *Streptococcus constellatus* (10.6%), and *Klebsiella pneumoniae* (10.6%). Other isolates included *Pseudomonas aeruginosa*, *Staphylococcus aureus*, *Serratia marcescens*, *Peptostreptococcus anaerobius*, and *Streptococcus anginosus*. In group comparisons, *S. constellatus* and *K. pneumoniae* were significantly more frequent in Group B (*p* = 0.028 ^c^ and *p* = 0.026 ^c^, respectively), whereas *p*. aeruginosa was observed only in Group A (11.5% vs. 0.0%, *p* = 0.026 ^c^).

Polymicrobial infections were observed in 14 patients (29.8%), more often in Group B than in Group A (57.1% vs. 7.7%), but this difference was not statistically significant (*p* = 1.000 ^c^). Antibiotic-resistant organisms were identified in 14 patients (29.8%) and were significantly more common in Group B compared to Group A (52.4% vs. 7.7%, *p* < 0.001). Fungal cultures were positive in 16 patients (34.0%), all of whom belonged to the non-OMS group (61.5%), while no positive fungal cultures were identified in the OMS group (0%) (*p* < 0.001). The detected isolates were mainly *Candida* species, regarded as colonizing organisms rather than invasive pathogens, indicating that a positive fungal culture should not be considered a direct indication for OMS intervention (Table 2).

**Figure 1:**
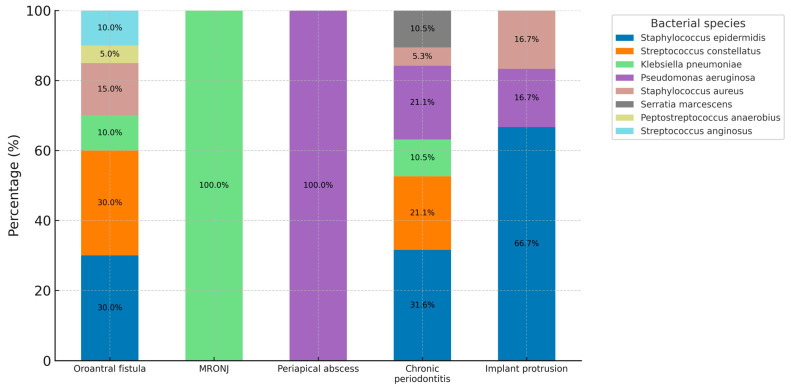
Overall distribution of bacterial species isolated from patients with ODS.

**Figure 2:**
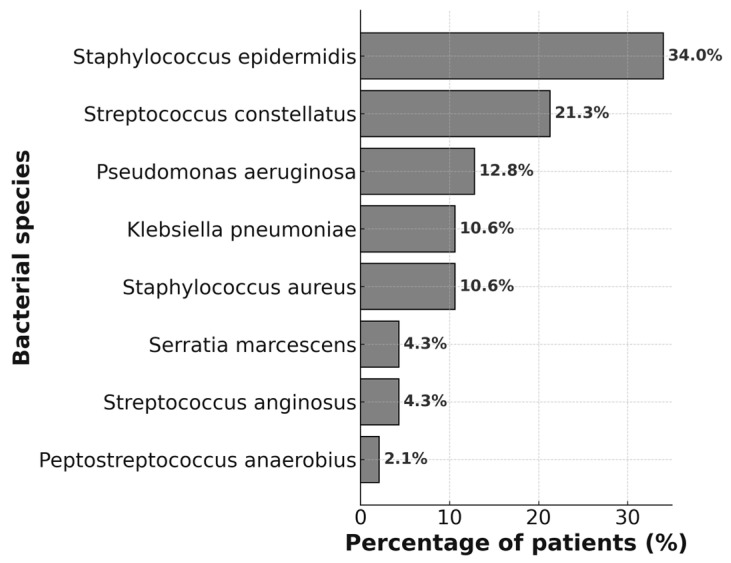
Distribution of bacterial species by dental diagnosis. Each bar represents the relative frequency of bacterial species isolated from patients within each dental diagnosis group. Percentages are shown inside the bars. *Staphylococcus epidermidis*, *Streptococcus constellatus*, and Staphylococcus aureus were the most frequently identified pathogens across diagnoses.

The overall distribution of bacterial isolates is shown in Figure 1 and Figure 2. *Staphylococcus epidermidis* was the most frequently identified species, accounting for 34.0% of cases. *Streptococcus constellatus* was the second most common isolate (21.3%), followed by *Pseudomonas aeruginosa* (12.8%), *Klebsiella pneumoniae* (10.6%), and *Staphylococcus aureus* (10.6%). Less frequently detected organisms included *Serratia marcescens* (4.3%), *Peptostreptococcus anaerobius* (2.1%), and *Streptococcus anginosus* (4.3%).

Across dental diagnoses, *S. epidermidis* predominated in cases with oroantral fistula and implant protrusion into the maxillary sinus, whereas *S. constellatus* was most common in periapical abscesses and odontogenic cysts. Gram-negative species such as *K. pneumoniae* and *P. aeruginosa* were more frequently detected in chronic periodontitis. Detailed distributions by diagnosis and management are provided in Figure 1 and Figure 2. Among patients classified as having periodontitis-related ODS, all demonstrated CT evidence of cortical bone discontinuity or sinus-floor elevation, and several had coexisting periapical radiolucencies. No cases of isolated periodontitis without bony involvement were include. Fungal culture was positive in 16 patients (34.0%), all within the non-OMS group (61.5%), while no OMS patient showed fungal growth (0%) (*p* < 0.001). Positive fungal culture was more common among older individuals and those with longer symptom duration, and clinically it was more often associated with foul odor, purulent rhinorrhea, or postnasal drip. When examined by dental pathology, fungal growth was observed in chronic periodontitis (7 cases), oroantral fistula (3 cases), and in some implant-protrusion cases (5 cases)—all occurring in the non-OMS cohort. Positive fungal culture showed no association with antibiotic resistance or polymicrobial infection. Accordingly, a positive fungal culture should not be considered a direct indication for OMS intervention.

**Figure 3:**
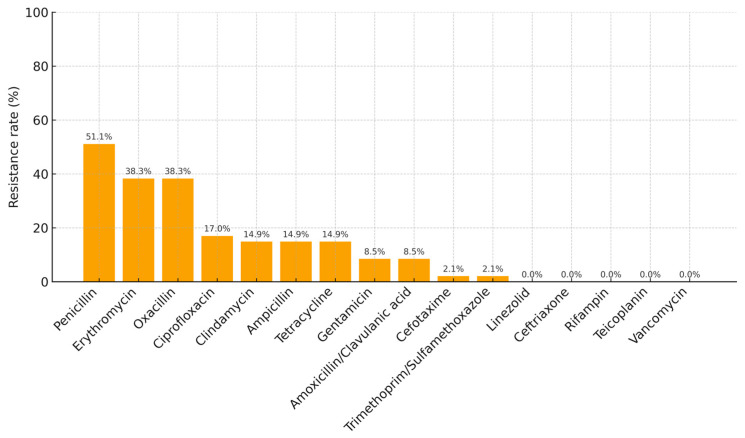
Antibiotic-specific resistance rates are presented in order of frequency.

The antibiotic resistance profiles of bacterial isolates are summarized in Table 3 and Figure 3. Penicillin showed the highest resistance rate (51.1%), followed by erythromycin (38.3%) and oxacillin (38.3%). Resistance to ciprofloxacin and clindamycin was observed in 17.0% and 14.9% of isolates, respectively. Resistance rates to ampicillin and tetracycline were 14.9% each, whereas gentamicin and amoxicillin/clavulanic acid exhibited resistance in 8.5% of cases. Resistance to cefotaxime, trimethoprim/sulfamethoxazole, and linezolid was detected in only one or two patients (≤2.1%). No resistance was observed for ceftriaxone, rifampin, teicoplanin, or vancomycin (Table 3).

Multivariable logistic regression analysis identified specific dental diagnoses and antibiotic resistance as independent predictors of OMS intervention (Table 4). Oroantral fistula was significantly associated with higher odds of OMS intervention (OR, 4.25; 95% CI, 1.10–16.4; *p* = 0.019), whereas implant protrusion was observed only in Group A and showed an inverse association with OMS intervention (*p* = 0.020). The presence of antibiotic-resistant organisms was also an independent predictor (OR, 3.12; 95% CI, 0.55–17.8; *p* = 0.011). In contrast, hypertension and age were not significantly associated with OMS intervention.

All patients achieved full recovery without treatment failure; therefore, outcome comparison between groups was not feasible.

## 4. Discussion

This study identified key clinical and microbiological predictors associated with the need for oral and maxillofacial surgical (OMS) intervention in odontogenic sinusitis (ODS). Specifically, oroantral fistula and antibiotic-resistant infection emerged as independent predictors of OMS intervention, while fungal colonization occurred exclusively in the non-OMS intervention group. These findings underscore the importance of early recognition, multidisciplinary assessment, and careful interpretation of fungal cultures in the management of ODS. Beyond these principal findings, our investigation further delineated the clinical and microbiological spectrum of ODS cases requiring OMS involvement. In line with previous literature, ODS was frequently associated with polymicrobial infections dominated by anaerobes, with *Staphylococcus epidermidis* and *Streptococcus constellatus* among the most common isolates [10,11,12]. Compared with rhinogenic CRS—where *Streptococcus pneumoniae*, *Haemophilus influenzae*, and *Moraxella catarrhalis* are more typical pathogens—the predominance of organisms such as *Klebsiella pneumoniae*, *Staphylococcus aureus*, and viridans streptococci in ODS highlights the importance of targeted diagnostic and therapeutic strategies [11,12,13]. A notable finding was that antibiotic-resistant organisms were significantly more prevalent in patients requiring OMS procedures. Previous studies have emphasized that conventional culture-based diagnostics are often limited in detecting anaerobic bacteria, delaying optimal therapy [12]. Our findings highlight the importance of early recognition of resistance patterns, as targeted antibiotic therapy remains central to the management of odontogenic infections. Clinically, this suggests that suspected or confirmed resistance may warrant earlier referral to OMS, incorporation into risk-stratification workflows for patients with implant-related disease, periapical pathology, or chronic periodontitis, and more judicious selection of empirical antibiotics aligned with likely polymicrobial, anaerobe-dominant flora [15]. In terms of clinical presentation, foul odor and purulent rhinorrhea were common symptoms, consistent with prior reports identifying these as relatively specific for ODS compared with CRS [8,9]. However, dental symptoms such as gingival swelling or pain were not universal, highlighting the risk of underdiagnosis if dental evaluation is not systematically performed. This observation supports current guidelines recommending thorough dental assessment in all cases of unilateral maxillary sinusitis [14]. From a surgical perspective, our results demonstrate that the presence of oroantral fistula and specific dental pathologies, together with resistant organisms, were independent predictors for OMS intervention. These findings align with earlier observations that a multidisciplinary approach involving otolaryngologists and maxillofacial surgeons has been associated with improved outcomes in ODS [17]. As emphasized earlier, antibiotics alone cannot eradicate the odontogenic source of infection; definitive treatment requires elimination of the dental focus through coordinated dental or surgical intervention. In addition to otolaryngologists and maxillofacial surgeons, dentists and endodontists are integral members of the multidisciplinary team managing ODS. Early recognition and treatment of odontogenic infections through root canal therapy can eradicate the dental source in cases of pulpitis or periapical pathology, thereby preventing sinus extension and preserving the affected tooth. Successful endodontic management may thus obviate the need for surgical extraction or OMS intervention in select patients, underscoring the importance of timely dental evaluation and coordinated care.

Fungal colonization represented one of the most distinctive findings of this study. In our cohort, positive fungal cultures were observed exclusively in the non-OMS group (61.5%) and were absent in all OMS patients. This pattern suggests that fungal growth in ODS more often reflects colonization or the presence of a non-invasive fungal ball, rather than a true indication for maxillofacial intervention. Consistent with recent evidence linking dental implants with maxillary sinus fungus ball formation, positive fungal culture may occur in association with implant-related changes in the sinus floor. Importantly, such cases can frequently be managed effectively with endoscopic sinus surgery alone, without requiring additional oral and maxillofacial procedures [18]. Therefore, positive fungal cultures should be interpreted with caution and not regarded as independent determinants of OMS intervention, but rather assessed in the broader context of dental pathology, imaging findings, and clinical presentation. All patients in this study achieved full recovery following surgery, underscoring the efficacy of coordinated surgical management when both sinonasal and odontogenic sources are addressed. In addition to these clinical outcomes, it is noteworthy that culture-based methods, although useful for identifying characteristic pathogens, have inherent limitations. The application of next-generation sequencing could provide a more comprehensive view of the ODS microbiome [12,19,20]. Recent studies have demonstrated that odontogenic infections are predominantly polymicrobial, with anaerobes such as *Fusobacterium, Prevotella*, and *Porphyromonas* playing a major role [12,21,22,23,24,25]. They have also highlighted the frequent presence of genes resistant to commonly used agents such as clindamycin [26,27,28]. These findings support our observation that antibiotic resistance is a significant predictor of OMS intervention, underscoring the need for early recognition of high-risk patients and the consideration of advanced microbiological tools to guide therapy in the future. Compared with previous studies that have primarily focused on bacterial profiles or general treatment outcomes, our study systematically integrated dental pathology, microbiological findings, and treatment predictors. This comprehensive approach highlights the distinct role of oroantral fistula and implant protrusion, together with antibiotic resistance, as key determinants for OMS intervention. By combining dental and microbiological perspectives, our findings provide additional insight into the factors driving surgical decision-making and offer direct clinical relevance. Specifically, this integrative framework may help clinicians identify high-risk patients earlier, guide timely multidisciplinary referral, and optimize surgical planning. The present study has several limitations. Its retrospective design and single-center setting may limit generalizability, and the relatively small sample size precluded more detailed subgroup analysis. These limitations should be addressed by future multicenter prospective studies with larger sample sizes to validate and extend our findings.

## 5. Conclusions

This study shows that antibiotic resistance and specific dental pathologies, particularly oroantral fistula and implant protrusion, are independent risk factors for OMS intervention in odontogenic sinusitis. The recognition of these predictors can support early risk stratification and facilitate multidisciplinary surgical planning. Although all patients ultimately achieved resolution, the higher prevalence of resistant organisms in the OMS group emphasizes their clinical significance. Future multicenter, prospective studies using advanced diagnostic methods, including next-generation sequencing, are warranted to validate and extend these findings.

## Figures and Tables

**Table 1:** Patient characteristics and comparison according to OMS intervention.

		OMS Intervention		
	All Patients (%)	No (Group A)	Yes (Group B)	
	(*n* = 47)	(*n* = 26)	(*n* = 21)	*p*
Sex (male)				1.000
Male	20 (42.6%)	10 (38.5%)	10 (47.6%)	0.738
Female	27 (57.4%)	16 (61.5%)	11 (52.4%)	
Age (years)	59.5 ± 15.1	64.4 ± 14.8	53.5 ± 13.5	0.012 ^a^/0.009 ^b^
Underlying disease				
HTN	12 (25.5%)	10 (38.5%)	2 (9.5%)	0.020 ^c^
DM	14 (29.8%)	9 (34.6%)	5 (23.8%)	0.528
Osteoporosis	1 (2.1%)	1 (3.8%)	0 (0.0%)	1.000 ^c^
Cancer Hx	1 (2.1%)	1 (3.8%)	0 (0.0%)	1.000 ^c^
Hematologic disease	1 (2.1%)	1 (3.8%)	0 (0.0%)	1.000 ^c^
Symptom				
Cheek pain	1 (2.1%)	0 (0.0%)	1 (4.8%)	0.447 ^c^
Nasal obstruction	4 (8.5%)	4 (15.4%)	0 (0.0%)	0.117 ^c^
Purulent rhinorrhea	15 (31.9%)	7 (26.9%)	8 (38.1%)	0.533
Foul odor	10 (21.3%)	5 (19.2%)	5 (23.8%)	0.734
Postnasal drip	7 (14.9%)	6 (23.1%)	1 (4.8%)	0.112 ^c^
Gingival pain and swelling	7 (14.9%)	1 (3.8%)	6 (28.6%)	0.035 ^c^
Headache	1 (2.1%)	1 (3.8%)	0 (0.0%)	1.000 ^c^
CT abnormality (No Sx)	2 (4.3%)	2 (7.7%)	0 (0.0%)	0.495 ^c^
Symptom duration (weeks)	31.0 ± 54.8	36.8 ± 54.8	23.8 ± 55.3	0.427/0.146 ^b^
Dental Sx/Treatment Hx	39 (83.0%)	19 (73.1%)	20 (95.2%)	0.059 ^c^
Dental Problem				
No	7 (14.9%)	6 (23.1%)	1 (4.8%)	0.112 ^c^
Spontaneous tooth loss	4 (8.5%)	2 (7.7%)	2 (9.5%)	1.000 ^c^
Tooth extraction	13 (27.7%)	7 (26.9%)	6 (28.6%)	1.000
Dental implant	13 (27.7%)	7 (26.9%)	6 (28.6%)	1.000
Tooth mobility	10 (21.3%)	4 (15.4%)	6 (28.6%)	0.306 ^c^
Dental Diagnosis				
Oroantral fistula	20 (42.6%)	6 (23.1%)	14 (66.7%)	0.007
MRONJ	1 (2.1%)	0 (0.0%)	1 (4.8%)	0.447 ^c^
Periapical abscess	1 (2.1%)	1 (3.8%)	0 (0.0%)	1.000 ^c^
Chronic periodontitis	19 (40.4%)	13 (50.0%)	6 (28.6%)	0.234
Implant protrusion	6 (12.8%)	6 (23.1%)	0 (0.0%)	0.026 ^c^

^a^ Welch’s *t*-test; ^b^ Mann–Whitney *U* test; ^c^ Fisher’s exact test applied when the expected cell count was <5. After false discovery rate (FDR) correction for multiple testing, no variable remained statistically significant. All other categorical variables were analyzed using the chi-square test. (HTN, hypertension; DM, diabetes mellitus; MRONJ, medication-related osteonecrosis of the jaw; OMS, oral and maxillofacial surgery).

**Table 2:** Microbiological findings in patients with ODS according to OMS intervention.

		OMS Intervention		
	All Patients (%)	No (Group A)	Yes (Group B)	
	(*n* = 47)	(*n* = 26)	(*n* = 21)	*p*
Bacterial species				
*Staphylococcus epidermidis*	16 (34.0%)	11 (42.3%)	5 (23.8%)	0.307
*Streptococcus constellatus*	10 (21.3%)	2 (7.7%)	8 (38.1%)	0.028 ^c^
*Klebsiella pneumoniae*	5 (10.6%)	3 (11.5%)	2 (9.5%)	1.000 ^c^
*Pseudomonas aeruginosa*	6 (12.8%)	6 (23.1%)	0 (0.0%)	0.026 ^c^
*Staphylococcus aureus*	5 (10.6%)	3 (11.5%)	2 (9.5%)	1.000 ^c^
*Serratia marcescens*	2 (4.3%)	1 (3.8%)	1 (4.8%)	1.000 ^c^
*Peptostreptococcus anaerobius*	1 (2.1%)	0 (0.0%)	1 (4.8%)	0.447 ^c^
*Streptococcus anginosus*	2 (4.3%)	0 (0.0%)	2 (9.5%)	0.194 ^c^
Polymicrobial infection				
Yes	14 (29.8%)	2 (7.7%)	12 (57.1%)	1.000 ^c^
No	33 (70.2%)	24 (92.3%)	9 (42.9%)	1.000 ^c^
Antibiotic resistance				
Resistant	14 (29.8%)	2 (7.7%)	12 (57.1%)	<0.001 ^c^
Non-resistant	33 (70.2%)	24 (92.3%)	9 (42.9%)	<0.001 ^c^
Fungal culture				
Positive	16 (34.0%)	16 (61.5%)	0 (0.0%)	<0.001 ^c^
Negative	31 (34.0%)	10 (38.5%)	21 (100.0%)	<0.001 ^c^

Distribution of bacterial species, antibiotic resistance, polymicrobial infection, and fungal culture results in patients with ODS, compared between those who did and did not undergo OMS intervention. Values are presented as number (%); ^c^ Fisher’s exact test.

**Table 3:** Antibiotic resistance profile of bacterial isolates.

Antibiotic	Resistant n (%)	Sensitive n (%)
Penicillin	24 (51.1%)	23 (48.9%)
Erythromycin	18 (38.3%)	29 (61.7%)
Oxacillin	18 (38.3%)	29 (61.7%)
Ciprofloxacin	8 (17.0%)	39 (83.0%)
Clindamycin	7 (14.9%)	40 (85.1%)
Ampicillin	7 (14.9%)	40 (85.1%)
Tetracycline	7 (14.9%)	40 (85.1%)
Gentamicin	4 (8.5%)	43 (91.5%)
Amoxicillin/Clavulanic acid	4 (8.5%)	43 (91.5%)
Cefotaxime	1 (2.1%)	46 (97.9%)
Trimethoprim/Sulfamethoxazole	1 (2.1%)	46 (97.9%)
Linezolid	0 (0.0%)	47 (100.0%)
Ceftriaxone	0 (0.0%)	47 (100.0%)
Rifampin	0 (0.0%)	47 (100.0%)
Teicoplanin	0 (0.0%)	47 (100.0%)
Vancomycin	0 (0.0%)	47 (100.0%)

Penicillin, erythromycin, oxacillin, ciprofloxacin and clindamycin showed the highest resistance rates, whereas vancomycin, Teicoplanin, Rifampin, ceftriaxone and linezolid demonstrated the lowest. Values are presented as number (%).

**Table 4:** Multivariate logistic regression analysis for predictors of OMS intervention.

Predictor	OR (95% CI)	*p*-Value
Age (years)	0.95 (0.90–1.01)	0.094
Hypertension	0.07 (0.01–1.10)	0.200
Gingival pain and swelling	5.40 (0.61–47.7)	0.130
Antibiotic resistance (any)	3.12 (0.55–17.8)	0.011 *
Fungal culture (positive)	0.01 (0.00–0.12)	<0.011 *
Polymicrobial infection	2.87 (0.46–17.9)	0.260
Oroantral fistula	4.25 (1.10–16.4)	0.019 *
Implant protrusion to sinus	0.00 (NA–NA)	0.020 *

Logistic regression identified oroantral fistula (*p* = 0.019), implant protrusion (*p* = 0.020), and antibiotic resistance (*p* = 0.011) as independent predictors of OMS intervention. The model showed good discrimination (AUC = 0.902) and calibration (Hosmer–Lemeshow test, *p* = 0.247), supporting its appropriateness for identifying predictors of OMS intervention. Sensitivity analysis using ridge-penalized logistic regression yielded nearly identical coefficient estimates and directionality of associations (AUC = 0.901, Hosmer–Lemeshow *p* = 0.312), confirming that the identified predictors were robust and not affected by small-sample bias or quasi-separation. * Statistically significant at *p* < 0.05.

## Data Availability

The data presented in this study are available on request from the corresponding author. The data are not publicly available due to privacy and ethical restrictions.

## Abbreviations

The following abbreviations are used in this manuscript:

CRS	Chronic rhinosinusitis
CT	Computed tomography
ODS	Odontogenic sinusitis
OMS	Oral and maxillofacial surgery
OAF	Oroantral fistula
MRONJ	Medication-related osteonecrosis of the jaw
HTN	Hypertension
DM	Diabetes mellitus
CLSI	Clinical and Laboratory Standards Institute
ESS	Endoscopic sinus surgery
AUC	Area under the receiver-operating characteristic curve
